# Rapid Enzymatic Method for Pectin Methyl Esters Determination

**DOI:** 10.1155/2013/854763

**Published:** 2013-12-26

**Authors:** Lucyna Łękawska-Andrinopoulou, Efstathios G. Vasiliou, Dimitrios G. Georgakopoulos, Constantinos P. Yialouris, Constantinos A. Georgiou

**Affiliations:** ^1^Chemistry, Agricultural University of Athens, Iera Odos 75, 11855 Athens, Greece; ^2^Agricultural Microbiology, Agricultural University of Athens, Iera Odos 75, 11855 Athens, Greece; ^3^Informatics Laboratory, Agricultural University of Athens, Iera Odos 75, 11855 Athens, Greece

## Abstract

Pectin is a natural polysaccharide used in food and pharma industries. Pectin degree of methylation is an important parameter having significant influence on pectin applications. A rapid, fully automated, kinetic flow method for determination of pectin methyl esters has been developed. The method is based on a lab-made analyzer using the reverse flow-injection/stopped flow principle. Methanol is released from pectin by pectin methylesterase in the first mixing coil. Enzyme working solution is injected further downstream and it is mixed with pectin/pectin methylesterase stream in the second mixing coil. Methanol is oxidized by alcohol oxidase releasing formaldehyde and hydrogen peroxide. This reaction is coupled to horse radish peroxidase catalyzed reaction, which gives the colored product 4-N-(p-benzoquinoneimine)-antipyrine. Reaction rate is proportional to methanol concentration and it is followed using Ocean Optics USB 2000+ spectrophotometer. The analyzer is fully regulated by a lab written LabVIEW program. The detection limit was 1.47 mM with an analysis rate of 7 samples h^−1^. A paired *t*-test with results from manual method showed that the automated method results are equivalent to the manual method at the 95% confidence interval. The developed method is rapid and sustainable and it is the first application of flow analysis in pectin analysis.

## 1. Introduction

Pectin, a natural polysaccharide present in the cell walls of higher plants, is an important compound for food and pharma industries. The importance of the compound is related to its unique properties and the fact that it is biodegradable. The main raw materials from which commercial pectin is extracted are agricultural by-products, that is, citrus peel and apple pomace. The major applications of pectin in food products rely on its gelling and stabilizing properties. Pectin is commonly used for production of foodstuffs like jams, jellies, and marmalades as a gelling agent but also in acidified milk drinks, beverages, or confections as stabilizer [1]. The diversity of pectin applications and its beneficial health effects [2–4] are the reasons why this compound has received a lot of interest during recent years.

The gelling properties of pectin are a consequence of its structure. Pectin is primarily composed of *α*-(1-4)-linked D-galacturonic acid molecules, some of them being esterified with methanol. Degree of pectin methylation (DM) is defined as the percentage of methyl esterified carboxyl groups. DM 50% is the borderline between low-methoxyl (LM) and high-methoxyl (HM) pectins. DM influences the conditions that should be applied for pectin to form a gel. High-methoxyl pectins gel in the presence of sufficient amount of sugar and acid. Low-methoxyl pectins require divalent cations to form a gel structure. The ability of LM pectins to gel without sugar makes them especially suitable for dietetic, that is, low sugar foodstuffs [1]. The degree of methylation is related to setting times of pectins [5]. Based on the setting times, the most suitable pectin application can be chosen. For example, rapid setting pectins are perfectly suited for jams and marmalades, when settling or flotation of fruit pieces has to be avoided. In contrast, slow setting pectins are especially useful for production of jellies, when additional time for elimination of air bubbles before gelling is needed [1].

Pectins are used in the pharmaceutical industry in drug delivery systems [6]. Their applications greatly depend on the degree of methylation; for example, LM pectins are used in nasal preparations due to their ability to form gels in the presence of calcium cations [7]. DM also influences the drug release time from pectin-based matrix tablets when calcium formulations are used [8]. The application of pectin depends greatly on its degree of methylation. This is the reason why rapid and reliable methods for fast quantification of pectin methyl esters are of major interest and importance.

Pectin methyl esters can be hydrolyzed either by alkaline hydrolysis or enzymatically by pectin methylesterase. Released methanol can be quantified by means of GC [9, 10] and HPLC [11] or with alcohol oxidase [12]. When alcohol oxidase is used, the methanol content can be related to formaldehyde [13] or hydrogen peroxide concentration [14]. We present here a new automated flow injection-stopped flow method for determination of pectin methyl esters. A unique aspect of this method is that reagents are injected into flowing sample stream. This approach, called reverse flow injection (rFI), comes along with a variety of advantages, minimization of reagent consumption being the most common reason for choosing rFI mode. rFI is of interest when expensive reagents, like enzymes, are used, and also when the sample is in abundance, for example, when water is being analysed Moreover, rFI offers increased sensitivity. The sample is not injected and therefore there is no significant dispersion of the sample, improving sensitivity of the measurement. The limitations of rFI are related to sample throughput as it might be necessary to stop the system in order to change the sample or even to employ washing solution between the measurements. Nevertheless since the introduction of rFI in 1978, the number of publications using this approach is constantly increasing [15].

Flow methods are a well-established branch of analytical chemistry and their applications in quality assurance of food compounds [16] or pharmaceuticals [17] offer a rapid and sustainable alternative to manual methods. Flow injection systems are automated, providing rapid procedures of increased precision by minimizing human intervention and errors. The testing environment is identical for all samples, further increasing precision. Through flow methods sustainability is brought into analytical work. The method presented here is a rapid and sustainable way for analysis of crucial parameter of pectins with low reagent and sample consumption as well as minimal waste generation. The use of enzymes minimizes amount of chemicals used in the analytical work. Enzymes are a natural, biodegradable, and environmentally friendly alternative to chemicals. The method is based on an easily constructed manifold, which can be assembled from readily available commercial parts.

Flow-injection systems for methanol determination have been reported in the literature [18–23]. However, the use of flow-injection for pectin analysis remains unexplored. The application of flow-injection methods for pectin analysis offers novel, green technologies, which are in agreement with current sustainability trends.

## 2. Materials and Methods

### 2.1. Reagents

Enzyme working solution (EWS) is used in the flow and comparison method: 1.2 U mL^−1^ alcohol oxidase (AOX) (EC 1.1.3.13), 46 U mL^−1^ horseradish peroxidase (HRP) (EC 1.11.1.7), 7.5 mM phenol, and 2.5 mM 4-aminoantipyrine in 0.1 M phosphate buffer. Enzymes were purchased from Sigma Aldrich. All other reagents were of analytical grade. EWS used in the kinetic study contained 1 U mL^−1^ AOX and 40 U mL^−1^ HRP. A commercial preparation of a recombinant *Aspergillus oryzae* pectin methylesterase (PME) (EC 3.1.1.11) (Novoshape, Novozymes, Bagsvaerd, Denmark) was a generous gift from Dr. Hans Sejr Olsen. This preparation was filtered before use.

### 2.2. Standards and Samples

A stock solution of 0.25 g 100 mL^−1^ pectin equivalent to 7.2 mM methanol concentration was prepared in deionized water using HM citrus pectin of 69.5% degree of esterification. Dilutions 0.02, 0.05, 0.09, 0.12, 0.14, 0.18, 0.22 and 0.25 g 100 mL^−1^ corresponding to 0.49, 1.4, 2.6, 3.4, 4.0, 5.2, 6.3, and 7.2 mM methanol were prepared from the stock solution. Methanol concentrations calculations were based on degree of esterification and galacturonic acid content measurements. Methanol standards of 0.25, 0.49, 0.74, 0.99, 1.24, 1.48, and 1.73 mM for comparison method were prepared in deionized water and stored at −20°C. The standards were thawed just before measurement. Methanol standards used for the kinetic study were prepared in 0.1 M phosphate buffer, pH 7.5.

Pectins were donated by CP Kelco ApS (Lille Skensved, Denmark) and by Dr. Karen Marie Søndergaard, DuPont NHIB Denmark ApS.

### 2.3. Kinetic Study

A kinetic study of the influence of temperature and pH on the EWS was performed. The effect of different AOX activities ranging from 0.5 U mL^−1^ to 2.5 U mL^−1^ was examined. Stability of the EWS upon 3 months storage in the fridge was studied. 0.5 mL of MeOH standard was placed in the quartz cuvette of Jasco V-550 spectrophotometer and 2.5 mL EWS was added. The time course measurement program was started immediately to monitor reaction rate at 505 nm for 2-3 minutes. Reagent blanks were subtracted from the analytical signal when necessary.

### 2.4. Comparison Method

For comparison method, pectin saponification and neutralization were performed as described by Klavons and Bennett (1986) [12]. The absorbance was measured exactly 10 minutes after EWS addition. EWS reagent and sample blank were measured and subtracted.

### 2.5. Flow Injection Analyzer

**Figure 1:**
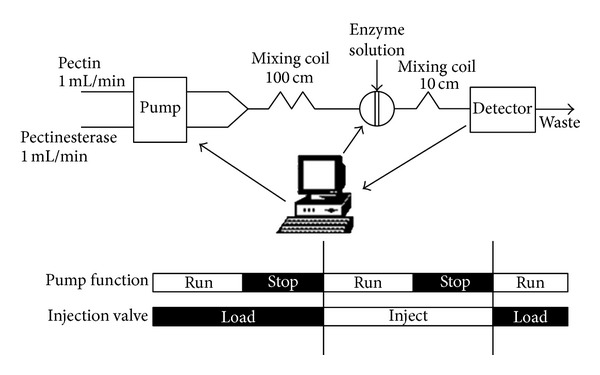
Manifold scheme and timing sequence of the analyzer.

The flow injection analyzer is depicted in Figure 1. Pectin is mixed in the first mixing coil with pectin methylesterase. First mixing coil was fixed at 100 cm, as this is sufficient length for methanol creation. Methanol is released as a result of action of the enzyme on methyl esterified groups of pectin (1):

(1) Pectin-COOCH3+H2O→Pectin  methylesterase  Pectin-COO−+H++CH3OH

(2) $$\begin{array}{c} \text{C}{\text{H}}_{3}\text{OH}+{\text{O}}_{2}\overset{\text{Alcohol oxidase}}{\to }\text{HCHO}+{\text{H}}_{2}{\text{O}}_{2} \end{array}$$

(3) 2H2O2+phenol+4-AAP→Horseradish  peroxidase  4-N-(p-benzoquinoneimine)-antipyrine+4H2O

EWS is injected further downstream. Methanol is oxidized by AOX releasing formaldehyde and hydrogen peroxide (2). This reaction is coupled to HRP catalyzed reaction, which gives the colored product 4-N-(p-benzoquinoneimine)-antipyrine (3). The reaction rate is proportional to methanol concentration and it is followed using Ocean Optics USB 2000+ spectrophotometer.

The analyzer is managed by a lab written program developed in LabVIEW which provides for data acquisition from the detector and control of the pump and injection valve. The program also controls the timing of the analyzer, as presented in Figure 1, displays raw data in the screen, and stores on the hard disc. Dedicated functions have been developed for controlling the load and inject positions of the Vici Valco injection valve and the stop and run positions of the Gilson Minipuls 3 peristaltic pump. Program runs on a personal computer incorporating the PCI-1760U Advantech card—used for controlling the peristaltic pump and the injection valve. Data acquisition from the spectrophotometer module was through the USB port controlled through LabVIEW module developed.

One minute is allowed for (1) to proceed; then EWS is injected. Nine seconds are allowed for the reaction mixture to reach the flow cell. Then the pump stops for five minutes to allow monitoring of (3). If needed, the operator has the choice to flush the system between successive measurements. PTFE tubing 0.8 mm i.d. was used.

### 2.6. Treatment of Data

The LabVIEW program acquires the whole spectrum: 189–1037 nm, every second. This creates a time-dependant data matrix. A separate, lab written program was used for extracting data at 505 nm. The program was built using Visual Basic and gets input from the file created by the LabVIEW program.

Reaction rates were calculated by regression analysis using the significant part of the data for each measurement.

## 3. Results and Discussion

**Figure 2:**
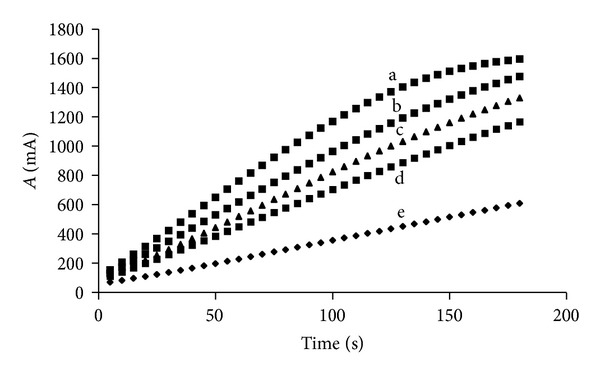
Alcohol oxidase (AOX) concentration influence on the analytical signal-reaction rate. a: AOX concentration: 2.5 U mL^−1^  (8.8 ± 0.2) × *t*  (s) + (198 ± 25), *r* = 0.99, b: AOX concentration: 2 U mL^−1^  (8.0 ± 0.1) × *t*  (s) + (13 ± 1) × 10, *r* = 0.997, c: AOX concentration: 1.5 U mL^−1^  (7.14 ± 0.05) × *t*  (s)+(89 ± 5), *r* = 0.9991, d: AOX concentration: 1 U mL^−1^  (6.12 ± 0.02) × *t*  (s)+(79 ± 2), *r* = 0.9997, and e: AOX concentration: 0.5 U mL^−1^  (3.12 ± 0.01) × *t*  (s)+(43 ± 1), *r* = 0.9998. Concentration of the methanol standard: 7.6 mM. Absorbance presented in milliabsorbance (mA).

A kinetic study of the reactions preceded the development of the manifold. To assure that the measured reaction rate is proportional to methanol concentration, the methanol oxidation reaction (2) must be the rate limiting step. On the other hand, the HRP catalyzed reaction (3) should be very fast. The influence of AOX concentration on the reaction curves was investigated for the methanol concentration as expected from the HM ~0.25 g 100 mL^−1^ pectin sample. When AOX activities higher than 1.5 U mL^−1^ are used, methanol is consumed within the monitoring time and linearity is lost towards the end of the measurement. In contrast, low AOX activities, such as 0.5 U mL^−1^, result in lower reaction rates, as shown in Figure 2. The activity of 1 U mL^−1^ was chosen as a tradeoff between high analytical signal and linearity.

**Figure 3:**
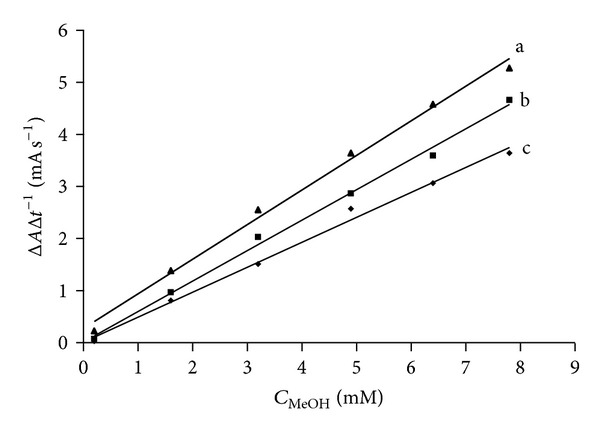
Calibration data obtained at different temperatures. a: 30°C Δ*A*Δ*t* ^−1^  (mA s^−1^) = (0.66 ± 0.0.03) × *C*  (mM)+(0.3 ± 0.1), *r* = 0.997, b: 25°C Δ*A*Δ*t* ^−1^  (mA s^−1^) = (0.58 ± 0.02) × *C*  (mM)+(0.01 ± 0.09), *r* = 0.998, and c: 20°C Δ*A*Δ*t* ^−1^  (mA s^−1^) = (0.48 ± 0.02) × *C*  (mM)+(0.01 ± 0.01), *r* = 0.997.

**Figure 4:**
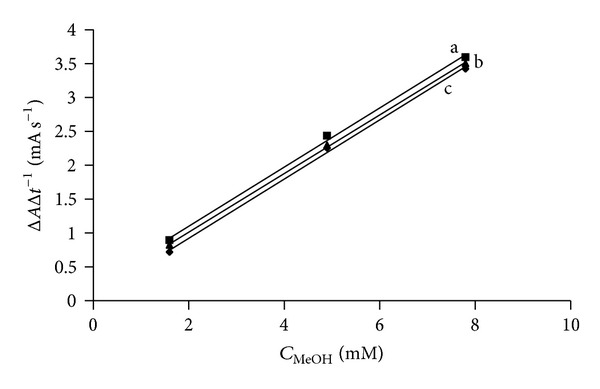
PH influence on the calibration curve. a: pH 7.0: Δ*A*Δ*t* ^−1^  (mA s^−1^) = (0.44 ± 0.02) × *C*  (mM)+(0.2 ± 0.1), *r* = 0.999, b: pH 7.5: Δ*A*Δ*t* ^−1^  (mA s^−1^) = (0.433 ± 0.009) × *C*  (mM)+(0.15 ± 0.05), *r* = 0.9998, and c: pH 6.5: Δ*A*Δ*t* ^−1^  (mA s^−1^) = (0.44 ± 0.02) × *C*  (mM)+(0.0 ± 0.1), *r* = 0.9991.

Robustness of the method strongly depends on the pH and temperature that affect enzyme activity.  Figure 3 shows the effect of the EWS temperature in the range 20–30°C. The reaction rate increases only 0.02 mA s^−1^°C^−1^. This proves that room temperature is appropriate for the automated method. Investigating the most optimal pH for the EWS was of special importance since enzymes in the EWS have different optimal pH: AOX: 7.5 [24] and HRP: 6–6.5 [25].  Figure 4 shows that pH changes of the enzyme working solution in the 6.5–7.5 range do not have any influence on the reaction rate. To summarize, the influence of temperature and pH on the reaction proved to be minor and the robustness of the method was confirmed. A long term stability study showed 19.5% and 30% loss of EWS activity after 48 and 99 days, respectively.

### 3.1. Flow Method Optimization

Univariate optimization was performed for the following parameters: (i) PME commercial preparation dilution, (ii) total flow rate, (iii) injection volume, (iv) preincubation time, the time allowed for methanol production (1), and (v) time allowed for the reaction mixture to reach the flow cell that is the second run time in Figure 1. Following parameter ranges were selected for the univariate optimization: total flow rate from 1 to 4 mL min^−1^, injection volume from 70 to 250 **μ**L, preincubation time from 2 to 60 s, and second run time from 3 to 11 s. The ranges were selected based on the limitations of fluidic systems.

The effect of dilution of the PME preparation was tested by diluting filtered Novoshape solution in deionized water up to 1 : 100. High PME activities increase the blank that reaches sample signal. This is probably due to glycerol contained in Novoshape as a stabilizer. AOX is not specific towards methanol and although it has higher affinity towards methanol, it probably also oxidizes glycerol. This is further supported by the observation that the signal of the blank was increasing along Novoshape concentration. To minimize reagent blank, higher Novoshape dilutions were assessed. It should be noted that, as expected, lower PME activities result in lower analytical signal. That is, although dilutions 1 : 60 and 1 : 100 show high signal to blank ratios (Table 1) they were rejected on the basis of 12 and 22% lower net analytical signal than for 1 : 20 dilution which was finally selected.

**Figure 5:**
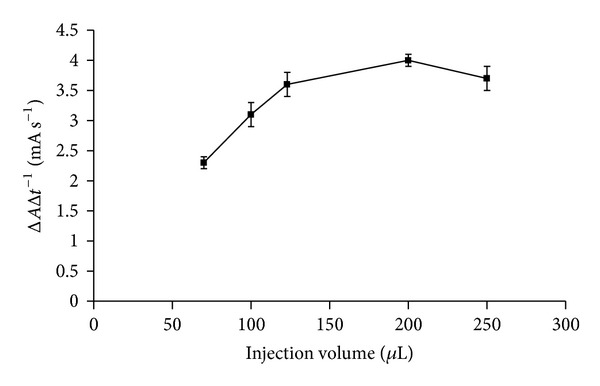
Injection volume effect on the reaction rate using pectin standard corresponding to 5.7 mM methanol.

The range of total flow rate considered for optimization was 1–4 mL min^−1^. A total flow rate of 2 mL min^−1^ resulted in the best mixing of reagents giving the highest analytical signal.  Figure 5 shows that signal increases along the increase of injected EWS volume in the range 70–123 *μ*L. 123 *μ*L was chosen as further increase up to 250 *μ*L does not affect significantly the analytical signal.

Preincubation time is related to PME action influencing methanol production in the first mixing coil.  Table 2 presents the effect of preincubation time on reaction rate in the range of 2 to 60 s. Although further increase of preincubation time is expected to result in increased signal, 60 s were chosen as a tradeoff between analytical run time and sensitivity. To shorten development time, 10 s preincubation time was used throughout optimization. A second run time used for allowing the reaction mixture to reach the flow cell (depicted in Figure 1) was varied from 3 to 11 s. The analytical signal increases along time, the maximum being observed at 9 s, and then it decreases. Interestingly, reagent blank observed during the kinetic study was fully eliminated.

Mixing coil placement after the injection valve facilitates mixing of EWS with pectin/PME stream. To assure that the reaction mixture is rapidly transported for monitoring in the flow cell, coil length should be the smallest possible. It should be noted that coils dampen peristaltic pump pulsations resulting in increased precision of flow analytical methods. Therefore and also due to the fact that (3) proceeds immediately a very short mixing coil, that is, 10 cm, was selected.

Particular attention has to be paid to avoid carry-over between samples and to diminish the effect of absorption/desorption processes on the manifold. These phenomena are particularly exaggerated in case of viscous samples such as pectin. Readings for the first daily calibration curve differ significantly from the following. First and two subsequent analyses of the standard corresponding to 1.4 mM methanol resulted in 1.03 ± 0.03, 1.81 ± 0.03, and 1.79 ± 0.03 mA s^−1^. This was solved by allowing 8 standard injections before actual measurements on startup, improving precision of 10 consecutive injections to 5.3% RSD.

**Figure 6:**
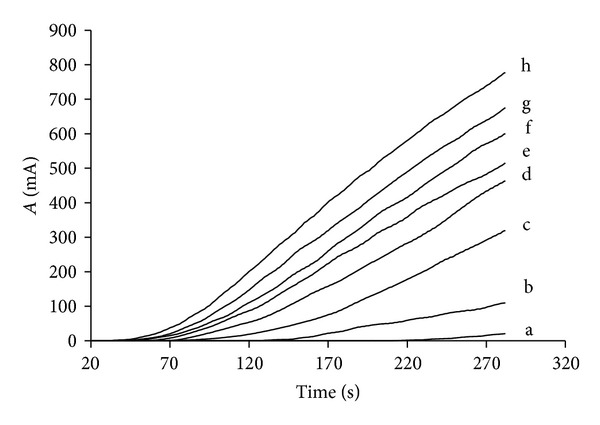
Stopped flow injection peaks for pectin calibration standards corresponding to following methanol concentrations: (a) 0 mM, (b) 0.49 mM, (c) 1.4 mM, (d) 2.6 mM, (e) 3.4 mM, (f) 4.0 mM, (g) 5.2 mM, (h) 6.3 mM, and (i) 7.2 mM. Absorbance presented in milliabsorbance (mA).

Typical readings acquired during the second stop of the pump illustrating the linearity of the method are shown in Figure 6. The presented data are smoothed by substituting values with the mean resulting from the previous 20 and following 20 readings. The calibration curve was reaction rate (mA s^−1^) = −0.0569*C* ^2^ + 0.8487*C* + 0.4622, *r* = 0.994.

Results from the analysis of samples in comparison to those acquired with the manual method are shown in Table 3. The detection limit calculated as LOD = 3.3 × *S*_intercept_/slope was found to be 1.47 mM. The analysis rate of the developed method is 7 samples h^−1^. Analysis time is shorter than in manual method where at least 30 minutes are required for saponification. To verify that the proposed method is equivalent to manual method a paired *t*-test was performed resulting in *t*_experimental_ 0.357 when *t*_theoretical_ is 3.18 for 3 d.f. at confidence level 95%, proving evidence that there is no significant difference between the two methods. For HM pectin the difference between two methods was insignificant, while for LM it was higher. This is probably due to the lower hydrolysis rate of LM pectins resulting from their lower methyl ester content. A remedy is to match samples with standards, using LM pectins for calibration when analyzing LM pectins. It is interesting to note that both reactions, catalyzed by PME and AOX, do not come to an end. Moreover, physical mixing of the injected EWS cocktail is also kinetic in nature, not coming to the steady state during the analytical run. These are clear differences from the manual method giving the opportunity of lower analysis time but also the challenge for selecting appropriate calibration standards.

In kinetic methods of analysis only the initial fraction of the reaction is monitored providing a fast alternative to time-consuming end point methods. Analytical parameter is not the absolute value of the signal but the rate of its change. This eliminates end point problems due to sample and/or reagent blank. With kinetic methods of analysis, interferences from slow reactions are insignificant while those resulting from fast reactions are eliminated by a short preincubation step where interfering species are consumed [26].

In order to increase the analysis rate and decrease the detection limit, the analyzer could be coupled to a more sensitive detection technique, that is, chemiluminescence, with which concentrations as low as 10 nM can be measured [27]. Use of chemiluminescence would allow higher dilution of samples and therefore the washing step could be eliminated and the monitoring time shortened.

## 4. Conclusions

This study reports the development of an automated flow method for determination of pectin methyl esters. Detection limit down to 1.47 mM was achieved at the analysis rate of 7 samples h^−1^. Paired *t*-test has shown no significant difference between results from the developed analyzer and manual offline method. The developed method is the first application of flow analysis in pectin quality assessment. Samples of increased viscosity such as olive oil or pectin need special attention when pumped in flow injection systems. Viscous samples can lead to carry-over effect between the samples. In order to avoid these problems additional washing step or saturation of the system prior to measurements might be necessary. Another solution could involve merging the analyzer with more sensitive detection method, for example, chemiluminescence. This would allow using higher dilutions of the sample introducing therefore lower viscosity. Further research should focus on the development of flow analyzer for galacturonic acid content, which together with the analyzer presented here would constitute a system of analyzers for the degree of methyl esterification.

## Supplementary Material

Data acquisition and control program flowchart.

## Figures and Tables

**Table 1:** Effect of pectin methylesterase commercial preparation activity on the analytical signal.

Pectinesterase dilution (v/v)	Ratio signal/blank
1 : 3	1.4
1 : 5	1.6
1 : 7	2.4
1 : 10	3.1
1 : 20	11.3
1 : 60	27.6
1 : 100	35.2

**Table 2:** Preincubation time effect on the reaction rate using pectin standard corresponding to 5.7 mM methyl esters.

Preincubation time (s)	Reaction rate ± SE (mA s^−1^)
2.0	2.4 ± 0.2
5.0	3.8 ± 0.1
10.0	4.9 ± 0.1
20.0	5.6 ± 0.1
30.0	6.4 ± 0.1
60.0	6.9 ± 0.1

**Table 3:** Comparison between the automated and the manual method.

Methanol content, mM		
	Automated method	Manual method
Sample 1	4.0	4.4
Sample 2	7.5	5.9
Sample 3	5.8	6.2
Sample 4	2.0	3.8
