# Supplementary figures and images for: Rapid Enzymatic Method for Pectin Methyl Esters Determination
Source: J Anal Methods Chem. 2013 Dec 26;2013:854763. doi: 10.1155/2013/854763 (PMC3888753; doi:10.1155/2013/854763)

## Data acquisition and control program flowchart

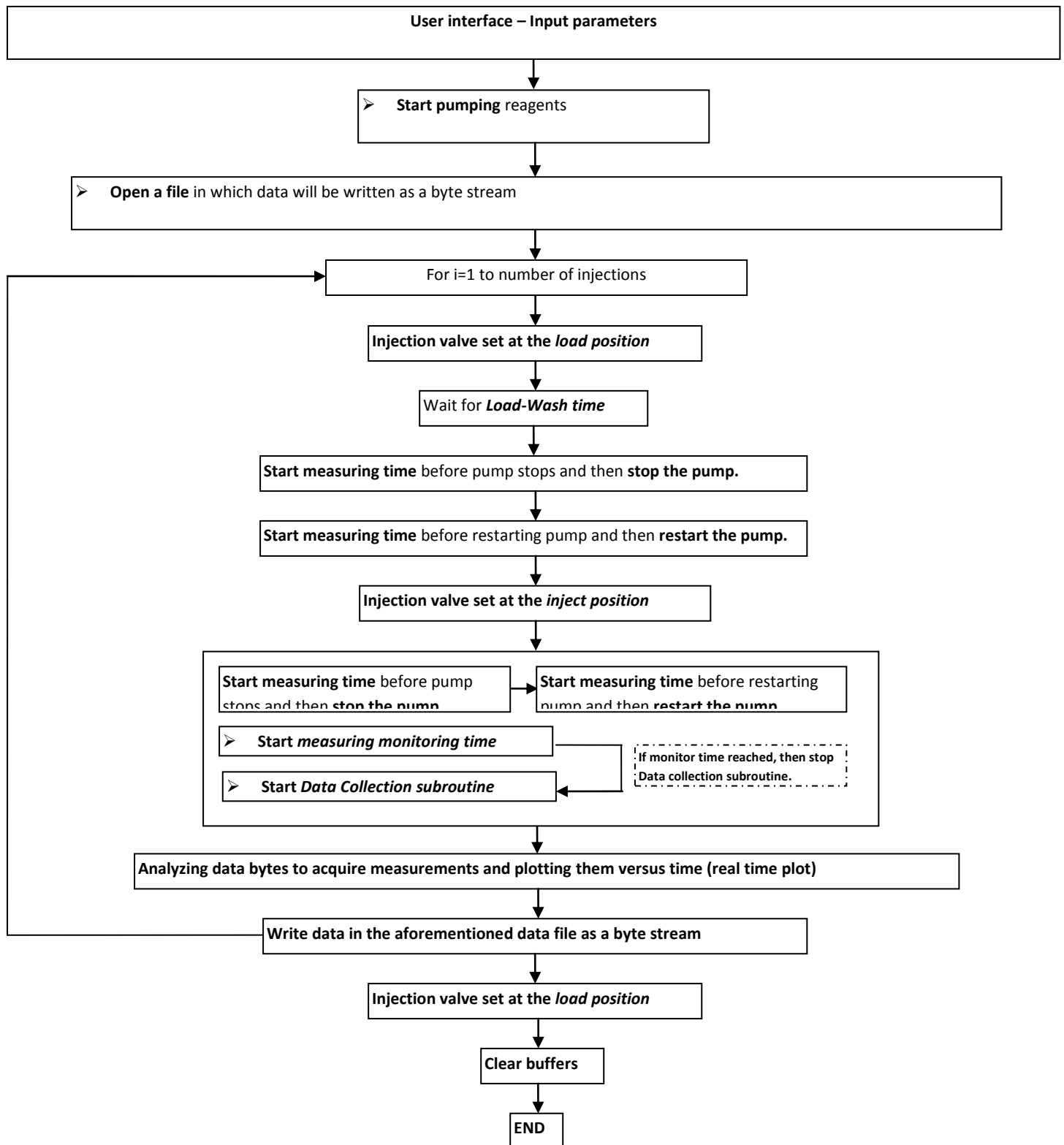

Supplement: Supplementary file 1 — Data acquisition and control program flowchart. [file 854763.f1.pdf]
